# Treadmill exercise improves motor deficits and partially normalizes electrophysiological properties of substantia nigra pars reticulata PV-lineage neurons in 6-OHDA-lesioned mice

**DOI:** 10.3389/fphys.2026.1896949

**Published:** 2026-09-11

**Authors:** Wanchun Xue, Jianhua Zhang, Zhiyu Chen

**Affiliations:** 1 College of Sports Science, Jishou University, Jishou, Hunan, China; 2 School of Physical Education and Health, Huaihua University, Huaihua, China; 3 Yulin Normal University, Yulin, China

**Keywords:** 6-hydroxydopamine, intrinsic excitability, parkinson’s disease, PV-lineage neurons, substantia nigra pars reticulata, treadmill exercise, whole-cell patch-clamp recording

## Abstract

**Objective:**

To examine whether treadmill exercise improves motor deficits and alters electrophysiological properties of substantia nigra pars reticulata (SNr) PV-lineage neurons in 6-OHDA-lesioned mice.

**Methods:**

Male Pvalb-Cre;Ai14 mice were assigned to Sham, 6-OHDA-lesioned sedentary (PD), and 6-OHDA-lesioned treadmill-exercise (PD+EX) groups. A unilateral 6-OHDA lesion model was established by right striatal injection of 6-OHDA. PD+EX mice underwent treadmill training for 30 min/day, 5 days/week for 6 weeks. Motor function was assessed using a 10-min open-field test and three 300-s rotarod trials. Lesion verification, PV Western blotting, and whole-cell patch-clamp recordings of SNr PV-lineage neurons were performed.

**Results:**

Six weeks of treadmill exercise partially improved open-field and rotarod performance in 6-OHDA-lesioned mice. Exercise was also accompanied by partial normalization of several electrophysiological abnormalities in lesioned-side SNr PV-lineage neurons, including input resistance, action potential threshold, evoked firing capacity, F–I gain, and firing adaptation. No significant group differences in regional PV protein abundance were observed.

**Conclusion:**

Treadmill exercise improved motor performance and was accompanied by partial normalization of the electrophysiological properties of SNr PV-lineage neurons in 6-OHDA-lesioned mice.

## 1 Introduction

Parkinson’s disease (PD) is a progressive neurodegenerative disorder characterized primarily by bradykinesia, muscular rigidity, resting tremor, and impaired postural control (Yang et al., 2022; Trevisan et al., 2024; Yalçin et al., 2025). The progressive loss of dopaminergic neurons in the substantia nigra pars compacta (SNpc) and the resulting depletion of dopamine in the nigrostriatal pathway are major pathological features of PD (Chen X. et al., 2023; Barmpa et al., 2026). However, PD-related motor impairment cannot be explained solely by dopamine deficiency. Dopaminergic denervation also induces extensive functional remodeling of the basal ganglia–thalamocortical and brainstem motor circuits, including changes in neuronal excitability, synaptic input, firing patterns, and network synchronization (Chen L. et al., 2023; Hofman et al., 2025; McLoughlin et al., 2025; Yoo et al., 2026). Therefore, investigating specific neuronal populations within basal ganglia output nuclei may help clarify the cellular mechanisms underlying abnormal motor output after dopamine depletion.

Pharmacological treatments, particularly levodopa and dopamine receptor agonists, remain important for controlling PD motor symptoms (Ferraiolo and Hermans, 2023; Plewnia et al., 2025; Leiter et al., 2026). However, these treatments do not stop the underlying neurodegenerative process. Their long-term effectiveness may also be limited by motor fluctuations, dyskinesia, reduced duration of benefit, and incomplete improvement of axial symptoms such as gait and postural instability (Di Luca et al., 2022; Roytman et al., 2023; Yan et al., 2025; Schnalke et al., 2026). Exercise is therefore used as an adjunctive non-pharmacological intervention rather than as a replacement for medication. Exercise can be repeatedly administered, contributes to physical conditioning and balance training, and may engage activity-dependent neural plasticity (Bennell et al., 2022; Behan et al., 2024; Hsu et al., 2025). These characteristics provide a practical rationale for investigating whether exercise modulates neural circuits that are not directly restored by dopaminergic medication.

The SNr is one of the principal output nuclei of the basal ganglia in rodents (Delgado-Zabalza et al., 2023; Thompson et al., 2026). Through its γ-aminobutyric acid-mediated projections to the motor thalamus, superior colliculus, and brainstem motor regions, the SNr participates in movement initiation, postural regulation, orienting behavior, and motor execution (Villalobos and Basso, 2022; Falasconi et al., 2025; Thompson et al., 2025). According to the classical basal ganglia model, dopamine depletion weakens direct-pathway activity and enhances indirect-pathway activity, thereby altering basal ganglia output and suppressing downstream motor-related structures (Bech et al., 2023; Aristieta et al., 2024). Nevertheless, SNr dysfunction in PD is unlikely to represent only a uniform increase in output. Dopamine depletion may also alter the intrinsic membrane properties, synaptic integration, input–output transformation, and firing organization of distinct SNr neuronal populations (Delgado-Zabalza et al., 2023). Although the SNpc is the classical site of dopaminergic neuron loss in PD and was assessed here for lesion verification, the SNr was selected for functional and electrophysiological analysis because it is a key downstream basal ganglia output nucleus involved in motor control.

The SNr is molecularly, anatomically, and functionally heterogeneous (Delgado-Zabalza et al., 2023; Mendelsohn et al., 2025). Different neuronal populations exhibit distinct spatial distributions, projection targets, and electrophysiological properties and may therefore contribute differently to motor control and action selection (Delgado-Zabalza et al., 2023; Mendelsohn et al., 2025; Yoshida and Hikosaka, 2025). Parvalbumin (PV)-related neurons constitute a prominent GABAergic population in the SNr and have been implicated in motor-related output pathways (Zheng et al., 2021; Delgado-Zabalza et al., 2023). Importantly, manipulation of PV-expressing SNr neurons has been reported to influence motor performance in parkinsonian mice, suggesting that this population may participate in the expression or modulation of PD-like motor dysfunction. Previous work has shown that unilateral 6-OHDA lesioning alters the intrinsic electrophysiological properties of SNr PV-expressing neurons and has implicated reduced NALCN-mediated sodium leak conductance in this dysfunction. However, whether exercise intervention can modify these cell-type-specific electrophysiological abnormalities remains unknown (Delgado-Zabalza et al., 2023).

Exercise has received increasing attention as an adjunctive non-pharmacological strategy for PD rehabilitation (Schootemeijer et al., 2020; Li et al., 2025; Luthra et al., 2025). Clinical and experimental evidence indicates that aerobic and treadmill exercise can improve locomotor activity, gait, balance, and motor coordination (Monir et al., 2020; Méndez-Martínez and Rodríguez-Grande, 2023; Peng et al., 2026). Potential mechanisms include modulation of neurotrophic signaling, synaptic plasticity, oxidative stress, neuroinflammation, neurotransmission, and activity within motor circuits (Zikereya et al., 2023; Kaagman et al., 2024; Tsai et al., 2025; Cayir et al., 2026). Nevertheless, most previous studies have focused on the nigrostriatal dopaminergic system, striatal molecular changes, or general behavioral outcomes. Whether treadmill exercise is accompanied by functional changes in a defined neuronal population within a basal ganglia output nucleus remains insufficiently understood.

Both 6-OHDA and 1-methyl-4-phenyl-1,2,3,6-tetrahydropyridine (MPTP) are widely used to model dopaminergic injury (Guo et al., 2020; Wasel and Freeman, 2020; Zhu, 2026). In the present study, unilateral intrastriatal 6-OHDA injection was selected because it produces a spatially controlled and lateralized nigrostriatal lesion that can be screened using apomorphine-induced rotational behavior (Konieczny et al., 2017; Rosa et al., 2020; Mendes-Pinheiro et al., 2021; Slézia et al., 2023). This approach also enables electrophysiological recordings to be consistently performed in the SNr ipsilateral to the lesion while using the contralateral hemisphere as an internal reference for histological lesion verification. In comparison, systemic MPTP exposure generally produces more bilateral dopaminergic injury and may show variability related to the administration protocol and animal susceptibility (Alam et al., 2017; Ji et al., 2019; Pathania et al., 2021). The unilateral 6-OHDA model was therefore more suitable for the present study, which focused on lesion-side motor-circuit changes and SNr neuronal electrophysiology.

Accordingly, the present study used Pvalb-Cre;Ai14 mice to establish a unilateral intrastriatal 6-OHDA lesion model and combined 6 weeks of treadmill exercise with behavioral testing, TH-based lesion verification, regional PV Western blot analysis, and ex vivo whole-cell patch-clamp recordings of tdTomato-labeled PV-lineage neurons in the lesioned-side SNr. We investigated whether treadmill exercise improved spontaneous locomotor activity, motor coordination, and balance in 6-OHDA-lesioned mice and whether behavioral improvement was accompanied by partial normalization of the basic membrane and evoked firing properties of SNr PV-lineage neurons.

## 2 Materials and methods

### 2.1 Animals

Male Pvalb-Cre;Ai14 mice were generated by crossing Pvalb-Cre mice (JAX stock no. 017320) with Ai14 reporter mice (Rosa26-CAG-LSL-tdTomato; JAX stock no. 007914).45 In these mice, Cre-dependent tdTomato fluorescence permits visual identification of cells belonging to the Pvalb-Cre lineage. Because lineage labeling does not necessarily indicate continued PV protein expression at the experimental endpoint, tdTomato-positive neurons in the SNr are referred to as PV-lineage neurons throughout this study.

Male mice entered the study at 5 weeks of age and weighed 18–22 g. This starting age was selected because the complete experimental protocol, including lesion induction, model verification, 6 weeks of treadmill training, and endpoint assessments, lasted approximately 8 weeks; therefore, the mice were approximately 13 weeks old and had reached young adulthood at the time of the final behavioral, histological, biochemical, and electrophysiological assessments. Only male mice were included to reduce variability potentially associated with estrous-cycle-related hormonal fluctuations in this exploratory study. This sex-specific design limits the generalizability of the findings to female mice. Mice were housed under specific pathogen-free conditions at 22 ± 2 °C and 50%–60% relative humidity under a 12-h light/dark cycle, with food and water available ad libitum. All experimental procedures were approved by the Biomedical Research Ethics Committee of Jishou University (approval no. JSDX-2026-0078) and were conducted in accordance with institutional guidelines for the care and use of laboratory animals.

### 2.2 Experimental grouping, animal flow, and sample sizes

Mice were assigned to the Sham, 6-OHDA-lesioned sedentary (PD), or 6-OHDA-lesioned treadmill-exercise (PD+EX) group. Thereafter, PD and PD+EX are used only as predefined group labels. Sham mice received a right striatal injection of vehicle. PD mice received a right striatal 6-OHDA lesion and remained sedentary. PD+EX mice received the same 6-OHDA lesion followed by treadmill exercise.

The Sham group contained 21 mice, with no postoperative deaths or exclusions. Initially, 25 mice in the PD group received 6-OHDA injection. One mouse died after surgery and two mice failed to meet the apomorphine-induced rotation criterion, resulting in 22 successfully lesioned mice. Twenty-one were used in the experimental modules, and one was retained as a reserve animal and was not included in data analysis. Thus, the overall modeling success rate in the PD group was 22/25 (88.0%).

Initially, 16 mice in the PD+EX group received 6-OHDA injection. One mouse died after surgery and one mouse failed to meet the rotation criterion, resulting in 14 successfully lesioned mice. Thirteen were used in the experimental modules, and one was retained as a reserve animal and was not included in data analysis. The overall modeling success rate in the PD+EX group was therefore 14/16 (87.5%).

Independent animal cohorts were used for different terminal experimental modules. The behavioral cohort contained five mice per group and was used for apomorphine-induced rotation, open-field testing, and rotarod testing. TH immunofluorescence used five independent mice per group from the Sham and PD groups. Striatal TH Western blot analysis used three independent mice per group from the Sham and PD groups. Nigral PV Western blot analysis used three independent mice per group from the Sham, PD, and PD+EX groups. Patch-clamp recordings used five independent mice per group. Animals used for terminal histological, Western blot, and acute-slice electrophysiological experiments were from separate cohorts and did not contribute to more than one terminal assay.

No formal *a priori* power calculation was performed. The sample sizes were selected on the basis of previous unilateral 6-OHDA, exercise, and SNr electrophysiological studies (Konieczny et al., 2017; Alam et al., 2017; Pathania et al., 2021; McElvain et al., 2021; Ji et al., 2019, 2023; Koketsu et al., 2021), together with the feasibility of obtaining technically qualified whole-cell recordings from genetically labeled SNr neurons. Because the animal sample sizes, particularly those used for Western blot analysis, were limited, the study was considered exploratory and the conclusions were interpreted cautiously.

### 2.3 Establishment of the unilateral 6-OHDA lesion model

Mice in the PD and PD+EX groups received a unilateral right striatal injection of 6-OHDA under stereotaxic guidance. Sham mice underwent the same surgical procedure but received vehicle.

Immediately before injection, 6-OHDA was dissolved in sterile saline containing 0.02% ascorbic acid at a concentration of 3 μg/μL (Konieczny et al., 2017; Pathania et al., 2021). The vehicle contained the same saline and ascorbic acid solution without 6-OHDA. Mice were anesthetized by intraperitoneal injection of 2,2,2-tribromoethanol (375 mg/kg; working concentration, 25 mg/mL). After an adequate depth of anesthesia was achieved, each mouse was fixed in a stereotaxic apparatus, the skull was exposed, and stereotaxic coordinates were determined relative to bregma.

The right striatum was targeted at anteroposterior +0.5 mm and mediolateral +2.0 mm, with injections performed at two dorsoventral depths of −3.0 and −3.8 mm (Alam et al., 2017; Ji et al., 2019). These two depths were selected to distribute the toxin across the dorsoventral extent of the right striatum while maintaining a unilateral lesion suitable for rotational screening and lesion-side SNr recordings.

At each dorsoventral depth, 1 μL of 6-OHDA solution was injected at 0.5 μL/min, producing a total dose of 6 μg per mouse. After each injection, the needle was left in place for 5 min and then slowly withdrawn to reduce reflux along the injection tract. Sham mice received the same volume of vehicle at the same coordinates. After surgery, the incision was sutured and routine postoperative care was provided.

This unilateral intrastriatal protocol was selected because it permits spatially controlled dopaminergic denervation, behavioral screening using drug-induced rotation, and consistent electrophysiological analysis of the SNr ipsilateral to the lesion (Konieczny et al., 2017; Ji et al., 2019; Alam et al., 2017; Pathania et al., 2021; McElvain et al., 2021).

### 2.4 Apomorphine-induced rotation and model identification

On day 7 after 6-OHDA injection, apomorphine-induced rotation was assessed in all lesioned mice. Apomorphine was prepared at 0.125 mg/mL and administered intraperitoneally at 0.5 mg/kg (McElvain et al., 2021). Beginning 5 min after injection, rotational behavior was recorded for 30 min.

The number of rotations toward the side contralateral to the lesion minus rotations toward the lesioned side was calculated. Mice exhibiting more than 120 net contralateral rotations during 30 min were considered successfully lesioned and were eligible for subsequent experiments. Animals failing to meet this criterion were excluded before treadmill intervention. At the experimental endpoint, unilateral dopaminergic injury was additionally confirmed in independent cohorts using TH immunofluorescence and striatal TH Western blot analysis.

### 2.5 Treadmill exercise protocol

Treadmill exercise began after model identification. All treadmill adaptation and formal training sessions were conducted during the light phase at approximately the same time each day. Mice in the PD+EX group first underwent 3 consecutive days of adaptation to the treadmill environment. Formal treadmill exercise was then performed for 6 weeks, from experimental weeks 2 to 7, at 10–12 m/min, 0° incline, 30 min/day, and 5 days/week.

A six-lane touchscreen motor-driven rodent treadmill was used (SNSEK, China; adjustable speed range 1–60 m/min and incline range 0°–30°). The electrical stimulation module remained switched off throughout adaptation and formal training. When a mouse temporarily stopped running, gentle manual guidance was used to encourage continued movement. No electrical shock was administered.

After each 30-min session, respiration, gait, and general condition were observed before the mouse was returned directly to its home cage. No medication, injection, electrical stimulation, or other additional treatment was administered after treadmill exercise. Sham and PD mice received comparable handling and contact during the same experimental period to reduce nonspecific effects related to handling.

### 2.6 Behavioral testing

Behavioral testing was conducted during the light phase in experimental week 8 after completion of treadmill exercise. All tests were performed at approximately the same time of day to minimize potential circadian influences on behavioral outcomes. Mice were transferred to the testing room and allowed to acclimate for at least 30 min. Tests were conducted under stable lighting and relatively quiet conditions. The apparatus was cleaned between animals to reduce olfactory interference.

#### 2.6.1 Open-field test

Each mouse was placed individually in the center of a 40 × 40 cm open-field arena and allowed to explore freely for 10 min. Total distance traveled and mean movement speed were obtained directly from the tracking software. Total distance traveled was used as the primary measure of spontaneous locomotor activity.

#### 2.6.2 Rotarod test

Motor coordination and balance were assessed using a RotarodtestA apparatus (Listenlite; five testing lanes, rod diameter 3 cm, lane width 6 cm, speed range 0–120 rpm) with Rotarodtest V1.0 software.

Before formal testing, mice underwent 2 consecutive days of rotarod pretraining, with three trials per day and an interval of at least 1 h between trials. During formal testing, the rotation speed increased from 4 to 40 rpm over 300 s. Latency to fall was recorded. Each mouse completed three formal trials, separated by sufficient rest, and the mean latency was used as the animal-level value for statistical analysis. The two-day pretraining period was used to familiarize the mice with the apparatus and testing procedure. Trials were separated by at least 1 h to minimize fatigue. Testing was paused or terminated if a mouse exhibited persistent immobility, repeated immediate falls, or obvious signs of fatigue or distress.

### 2.7 Acute brain slice preparation

After the corresponding experimental period, mice assigned to electrophysiological recording were deeply anesthetized by intraperitoneal injection of 2,2,2-tribromoethanol (375 mg/kg; working concentration, 25 mg/mL) and rapidly decapitated. The brain was immediately removed and placed in ice-cold sucrose-based cutting solution continuously bubbled with 95% O_2_ and 5% CO_2_.

Coronal slices containing the SNr were prepared at a thickness of 300 μm (Ji et al., 2023). The cutting solution contained the following components (in mM): sucrose 220, KCl 2.5, NaH_2_PO_4_ 1.25, NaHCO_3_ 25, glucose 20, ascorbic acid 0.4, sodium pyruvate 2, MgSO_4_ 6, and CaCl_2_ 0.5.

Slices were incubated in artificial cerebrospinal fluid at 34 °C for 30 min and subsequently allowed to recover at room temperature for at least 1 h. The recording solution contained the following components (in mM): NaCl 125, KCl 2.5, NaH_2_PO_4_ 1.25, MgSO_4_ 2, CaCl_2_ 2, NaHCO_3_ 25, glucose 20, ascorbic acid 0.4, and sodium pyruvate 2. All solutions were continuously bubbled with 95% O_2_ and 5% CO_2_.

### 2.8 Ex vivo whole-cell patch-clamp recording

Whole-cell patch-clamp recordings were performed at room temperature on tdTomato-labeled PV-lineage neurons in the SNr ipsilateral to the right striatal lesion (Koketsu et al., 2021). tdTomato-positive cells were identified using fluorescence microscopy, and recording electrodes were positioned using infrared differential interference contrast imaging.

The intracellular solution contained the following components (in mM): K-gluconate 130, KCl 5, phosphocreatine 5, HEPES 10, EGTA 0.5, Na_2_ATP 1, Na-GTP 0.3, and MgSO_4_ 2. The pH was adjusted to 7.2, and osmolarity was adjusted to approximately 290 mOsm. Recording electrodes had a resistance of 6–8 MΩ.

Only cells with stable seals, stable series resistance, and no apparent membrane-potential drift were included. Cells were excluded if series resistance exceeded 25 MΩ or changed by more than 20% during recording.

Resting membrane potential, membrane capacitance, input resistance, and action potential threshold were measured in current-clamp mode. Stepwise depolarizing currents ranging from 50 to 400 pA in 50-pA increments were injected for 1 s to evoke action potentials.51 Evoked firing outcomes included the action potential number at each current intensity, the action potential number at 400 pA, the frequency–current (F–I) curve slope, and the adaptation ratio during 400-pA stimulation. The F–I curve slope was derived from the relationship between injected current and action potential number across the 50–400-pA steps and was expressed as spikes/50 pA.

The adaptation ratio was calculated as the last interspike interval divided by the first interspike interval during the 400-pA current step. A higher ratio indicated greater firing adaptation.

A total of 9 cells from 5 Sham mice, 10 cells from 5 PD mice, and 9 cells from 5 PD+EX mice met the recording quality criteria. One to three cells were recorded from each mouse. To account for multiple cells being obtained from the same animal, cell-level measurements were averaged within each mouse. These animal-level averages, rather than individual cells, were used as independent observations in group-level statistical analyses.

### 2.9 TH immunofluorescence, qualitative PV/tdTomato colocalization imaging, and histological lesion verification

Mice assigned to histological analysis were deeply anesthetized by intraperitoneal injection of 2,2,2-tribromoethanol (375 mg/kg; working concentration, 25 mg/mL) and transcardially perfused with phosphate-buffered saline, followed by 4% paraformaldehyde. Brains were removed, post-fixed in 4% paraformaldehyde at 4 °C, dehydrated sequentially in 20% and 30% sucrose, embedded in optimal cutting temperature compound, and cut into 25-μm coronal sections.

Sections were blocked for 1 h at room temperature in phosphate-buffered saline containing 10% normal goat serum and 0.3% Triton X-100 and incubated overnight at 4 °C with chicken anti-TH primary antibody (ab76442, Abcam; 1:1000). After washing, sections were incubated for 1 h at room temperature in the dark with Alexa Fluor 488-conjugated donkey anti-chicken IgY (H+L) secondary antibody (A78948, Invitrogen; 1:1000).

Images were acquired using an LSM 980 laser scanning confocal microscope (Carl Zeiss, Germany). Striatal images were obtained using tile scanning and automatic stitching. The same anatomical regions, magnifications, fluorescence channels, laser power, detector gain, pinhole size, scan speed, resolution, and image-processing settings were used for all animals.

TH fluorescence intensity was measured in the injected/lesioned-side and contralateral striatum after background correction using an adjacent cortical region. The injected/lesioned-side striatal signal was expressed as a percentage of the contralateral signal. TH signal intensity in the SNpc was quantified using the same lesion-side-to-contralateral-side approach.

Fluorescence intensity was used because striatal TH immunoreactivity is predominantly present in dense dopaminergic fibers and terminals and is therefore not suitable for neuronal cell counting. SNpc TH intensity was used only as an additional lesion-verification measure and should not be interpreted as an unbiased stereological estimate of the number of surviving dopaminergic neurons. Because the brain sections were not collected using a systematic random-sampling protocol suitable for unbiased stereological counting, SNpc TH fluorescence intensity was used as a supplementary regional index of dopaminergic lesion severity rather than as an estimate of the absolute number of surviving TH-positive neurons.

TH immunofluorescence was performed only in the Sham and PD groups because this assay was designed to verify successful establishment of the 6-OHDA lesion rather than to determine whether treadmill exercise restored dopaminergic neurons or striatal innervation. Consequently, the present study does not draw conclusions regarding exercise-induced recovery of TH expression. To provide qualitative cellular and anatomical context for SNr PV-lineage neurons, a representative SNr section from a Pvalb-Cre;Ai14 mouse was processed for PV immunofluorescence. Sections were incubated overnight at 4 °C with rabbit anti-PV antibody (Parvalbumin [E8N2U] Rabbit Monoclonal Antibody, #80561, Cell Signaling Technology; 1:1000), followed by incubation for 1 h at room temperature in the dark with F(ab′)_2_-goat anti-rabbit IgG (H+L) cross-adsorbed secondary antibody conjugated to Alexa Fluor 488 (A-11070, Invitrogen, Thermo Fisher Scientific; 1:1000). Nuclei were counterstained with DAPI, while endogenous tdTomato fluorescence was retained. PV immunoreactivity was displayed in green, endogenous tdTomato fluorescence in red, and DAPI in blue. Images were acquired using an LSM 980 laser scanning confocal microscope. The merged image was used to illustrate qualitative colocalization between tdTomato-labeled PV-lineage cells and PV immunoreactivity within the SNr. This image was included only as representative qualitative evidence of colocalization and regional distribution and was not used for quantitative colocalization analysis, cell counting, or between-group comparisons.

### 2.10 Western blot analysis

Lesioned-side striatal tissue and lesioned-side nigral tissue containing the SNr were collected separately. Striatal tissue was used to measure TH protein as an additional verification of dopaminergic denervation. Nigral tissue containing the SNr was used to measure overall regional PV protein abundance.

**Figure 1:**
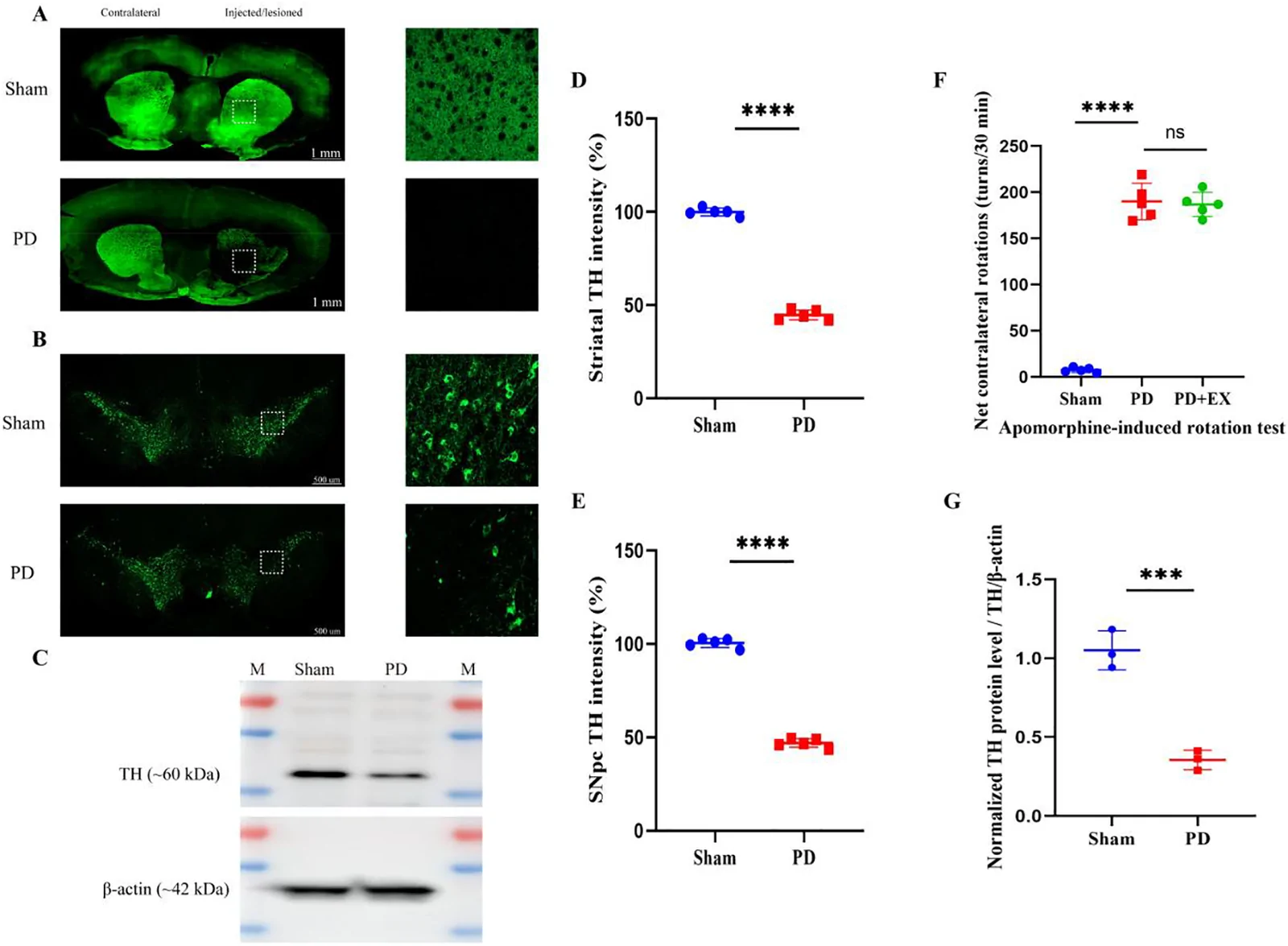
Verification of unilateral 6-OHDA-induced dopaminergic lesions. **(A)** Representative TH immunofluorescence images of the striatum, with higher-magnification views of the corresponding dashed-box regions shown at right. **(B)** Representative TH immunofluorescence images of the substantia nigra pars compacta (SNpc), with higher-magnification views of the corresponding dashed-box regions shown at right. “Contralateral” indicates the hemisphere contralateral to the injected side, whereas “Injected/lesioned” indicates the vehicle-injected hemisphere in Sham mice or the 6-OHDA-lesioned hemisphere in PD mice. Scale bars in the low-magnification images: 1 mm in **(A)** and 500 μm in **(B)**. **(C)** Representative complete membrane-strip images of TH and β-actin from lesioned-side striatal tissue, showing visible prestained molecular-weight markers and all analyzed sample lanes. The PVDF membrane was horizontally cut into molecular-weight-specific strips before antibody incubation. TH and β-actin were detected at approximately 60 and 42 kDa, respectively. **(D)** Injected/lesioned-side striatal TH fluorescence intensity expressed as a percentage of the contralateral-side signal. **(E)** Injected/lesioned-side SNpc TH intensity expressed as a percentage of the contralateral-side signal. **(F)** Net contralateral rotations during the 30-min apomorphine-induced rotation test performed before treadmill intervention. **(G)** Quantification of TH protein normalized to β-actin. TH immunofluorescence and TH Western blot analyses were performed only to verify the 6-OHDA lesion and were not used to assess exercise-induced restoration of dopaminergic innervation. Data are presented as mean ± SEM. TH immunofluorescence: n = 5 mice/group for Sham and PD; apomorphine-induced rotation: n = 5 mice/group for Sham, PD, and PD+EX; Western blot: n = 3 mice/group for Sham and PD. Each animal was treated as an independent statistical unit. TH immunofluorescence and Western blot data were analyzed using two-tailed unpaired t-tests. Rotation data were analyzed using one-way ANOVA followed by Holm-corrected pairwise comparisons. ***P < 0.001 and ****P < 0.0001; ns, not significant.

**Figure 2:**
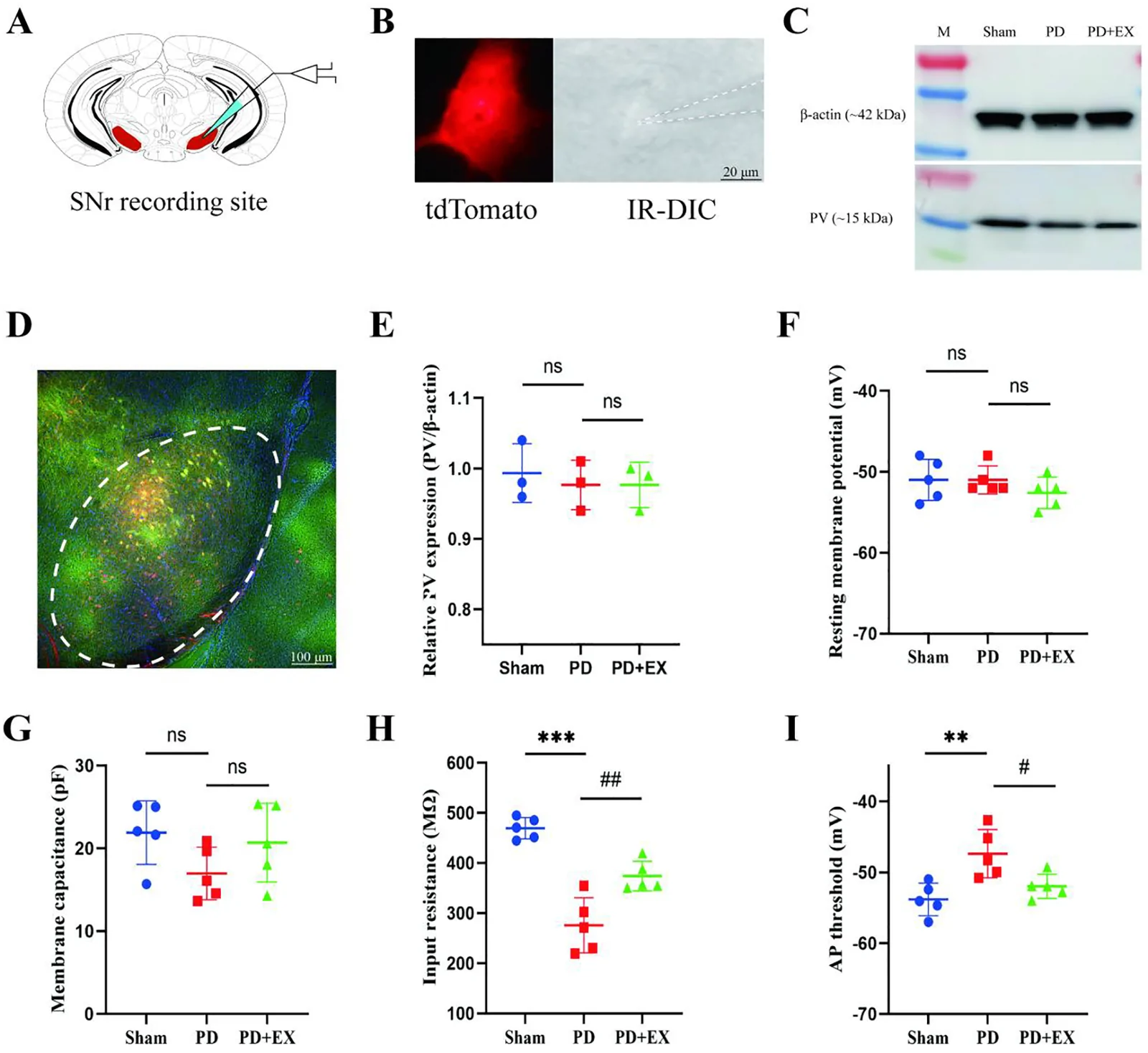
Treadmill exercise partially normalized the basic membrane properties of SNr PV-lineage neurons. **(A)** Schematic illustration of the substantia nigra pars reticulata (SNr) recording region. **(B)** Representative tdTomato-labeled SNr PV-lineage neuron and corresponding infrared differential interference contrast image. The dashed outline indicates the recorded cell body. Scale bar, 20 μm. **(C)** Representative complete membrane-strip images of β-actin and PV from lesioned-side nigral tissue containing the SNr, showing visible prestained molecular-weight markers and all analyzed sample lanes. The PVDF membrane was horizontally cut into molecular-weight-specific strips before antibody incubation. β-actin and PV were detected at approximately 42 and 15 kDa, respectively. **(D)** Representative merged immunofluorescence image showing qualitative colocalization between tdTomato-labeled PV-lineage cells and PV immunoreactivity within the SNr. PV immunoreactivity is shown in green, endogenous tdTomato fluorescence in red, and DAPI in blue. The dashed line indicates the approximate boundary of the SNr. Scale bar, 100 μm. This image is provided as representative qualitative colocalization evidence only and was not used for quantitative colocalization analysis, cell counting, or between-group comparisons. **(E)** Quantification of regional PV protein abundance normalized to β-actin. Regional PV Western blot analysis reflects overall PV protein abundance in the collected nigral tissue and does not specifically measure PV expression in individual tdTomato-labeled neurons or determine the number of PV-positive neurons. **(F)** Resting membrane potential. **(G)** Membrane capacitance. **(H)** Input resistance. **(I)** Action potential threshold. Data are presented as mean ± SEM. Western blot: n = 3 mice/group. Patch-clamp recordings included 9 cells from 5 Sham mice, 10 cells from 5 PD mice, and 9 cells from 5 PD+EX mice. Values obtained from multiple cells from the same mouse were averaged before group-level analysis, and the animal was treated as the independent statistical unit (n = 5 mice/group). Comparisons were performed using one-way ANOVA followed by Holm-corrected pairwise comparisons. Asterisks indicate comparisons with the Sham group, and number signs indicate comparisons with the PD group. *P or #P < 0.05; **P or ##P < 0.01; ***P or ###P < 0.001; ns, not significant.

Tissues were homogenized in RIPA buffer containing protease inhibitors. After centrifugation, the supernatant was collected and protein concentration was determined using a bicinchoninic acid assay. Equal amounts of protein were separated using SDS-PAGE and transferred to polyvinylidene fluoride membranes. Following transfer, the PVDF membranes were horizontally cut into molecular-weight-specific strips according to the positions of the prestained molecular-weight markers before antibody incubation. The complete available membrane-strip images containing visible molecular-weight markers and all analyzed sample lanes are presented in **Figures 1C**, **2C**.

Membranes were blocked with 5% nonfat milk for 1 h at room temperature and incubated overnight at 4 °C with rabbit anti-TH antibody (WL01820, Wanleibio; 1:1000), rabbit anti-PV antibody (#80561, Cell Signaling Technology; 1:1000), or mouse anti-β-actin antibody (HC201, TransGen Biotech; 1:5000).

After washing, membranes incubated with rabbit primary antibodies were treated with HRP-conjugated goat anti-rabbit IgG (31460, Invitrogen; 1:10000). Membranes incubated with mouse anti-β-actin were treated with HRP-conjugated goat anti-mouse IgG (A4416, Sigma-Aldrich; 1:10000). Protein bands were visualized using enhanced chemiluminescence, quantified using ImageJ, and normalized to β-actin.

Striatal TH Western blot analysis included three mice per group from the Sham and PD groups and was used only for lesion verification. Nigral PV Western blot analysis included three mice per group from the Sham, PD, and PD+EX groups.

Because PV was measured in bulk lesioned-side nigral tissue containing the SNr, these data reflect overall regional PV protein abundance. They do not specifically measure PV expression in isolated SNr PV-lineage neurons and cannot determine whether the number of PV-positive cells changed.

### 2.11 Statistical analysis

Data are presented as mean ± SEM. Behavioral, immunofluorescence, and Western blot analyses used the individual animal as the independent statistical unit. For patch-clamp experiments, measurements from multiple cells obtained from the same animal were first averaged, and the resulting animal mean was used as the independent observation. Thus, electrophysiological group comparisons were based on five animals per group, while the total numbers of recorded cells are also reported for sample-source transparency.

Three-group outcomes were analyzed using one-way analysis of variance followed, where appropriate, by pairwise comparisons with Holm correction. These outcomes included apomorphine-induced rotation, open-field distance, mean movement speed, rotarod latency, regional PV protein abundance, resting membrane potential, membrane capacitance, input resistance, action potential threshold, action potential number at 400 pA, F–I curve slope, and adaptation ratio.

Two-group TH lesion-verification outcomes were analyzed using two-tailed unpaired t-tests. The relationship between action potential number and current intensity was analyzed using two-way repeated-measures analysis of variance, with group as the between-subject factor and current intensity as the within-subject factor. For evoked-firing analyses, the primary comparisons were Sham versus PD and PD versus PD+EX. Holm-corrected pairwise comparisons were conducted at individual current intensities. Because this was an exploratory study with modest group sizes, current-specific comparisons were interpreted together with the omnibus group × current interaction and the overall consistency across related firing measures.

Effect sizes are reported as η² for one-way analysis of variance, partial η² for repeated-measures analysis, and Cohen’s d for two-group t-tests. Test statistics, degrees of freedom, and exact P values are reported in the Results. P < 0.05 was considered statistically significant.

## 3 Results

### 3.1 Experimental design, animal flow, and verification of the unilateral 6-OHDA lesion

**Figure 3:**
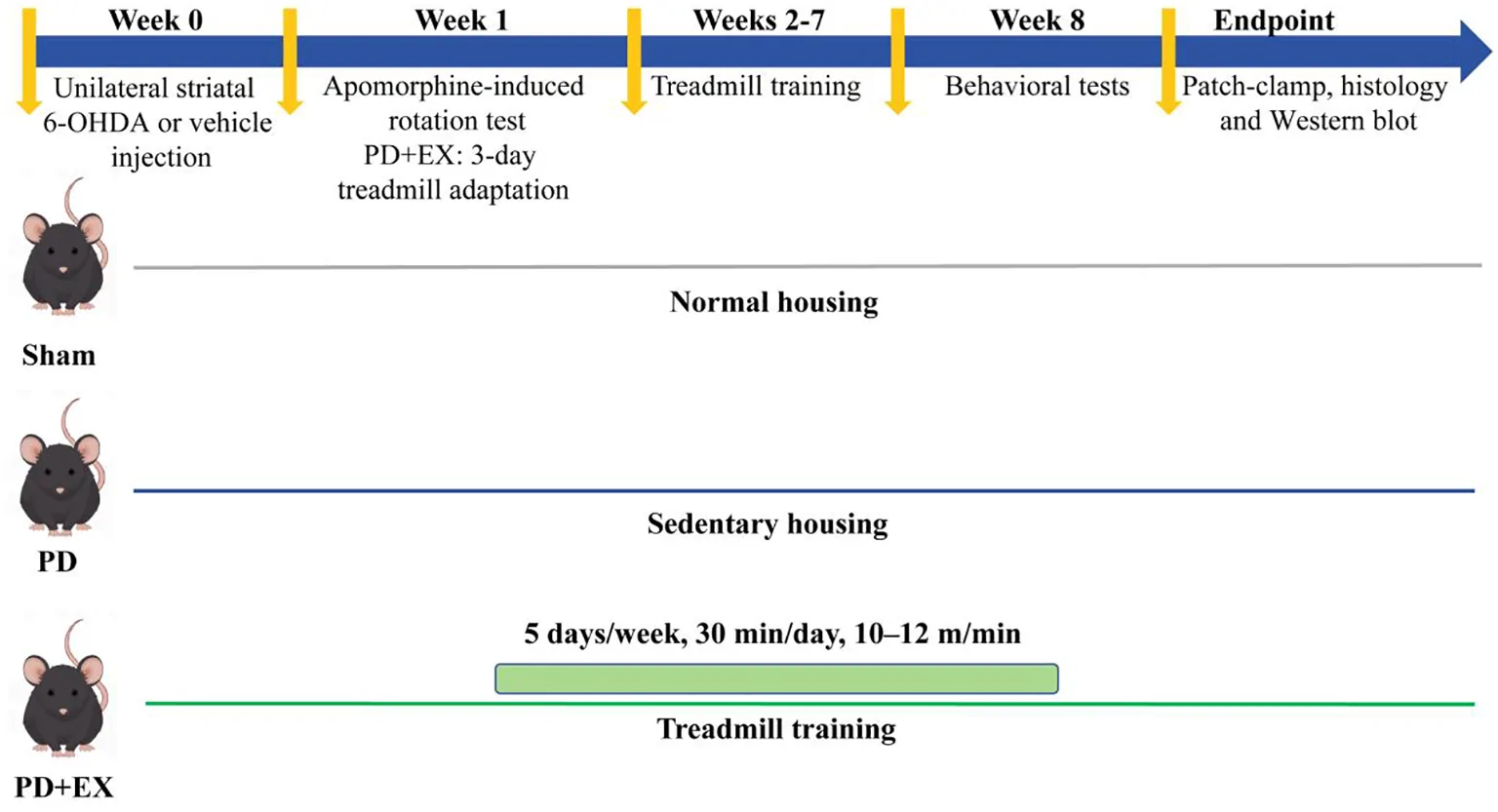
Experimental design and treadmill exercise protocol. Pvalb-Cre;Ai14 mice received a unilateral right striatal injection of 6-OHDA or vehicle at week 0. On day 7 after injection, apomorphine-induced rotation was assessed to identify successfully lesioned mice. Mice in the PD+EX group subsequently underwent 3 days of treadmill adaptation followed by 6 weeks of formal treadmill exercise from weeks 2 to 7 (10–12 m/min, 0° incline, 30 min/day, 5 days/week). Open-field and rotarod tests were performed in week 8. Tissue collection or ex vivo whole-cell patch-clamp recording was subsequently performed using independent animal cohorts according to the experimental purpose. Mice in the Sham and PD groups received comparable handling during the intervention period.

The overall experimental design is shown in **Figure 3**. Mice received a right striatal injection of 6-OHDA or vehicle at week 0. Apomorphine-induced rotation was assessed on day 7. After 3 days of treadmill adaptation, mice in the PD+EX group underwent 6 weeks of treadmill exercise. Behavioral testing was performed in week 8, followed by tissue collection or ex vivo electrophysiological recording using independent animal cohorts.

Of the 25 mice initially injected with 6-OHDA in the PD group, one died after surgery and two failed to meet the apomorphine-induced rotation criterion. Twenty-two mice were successfully lesioned, giving a modeling success rate of 88.0%. Twenty-one were used in the experimental modules, and one reserve animal was not included in data analysis.

Of the 16 mice initially injected with 6-OHDA in the PD+EX group, one died and one failed to meet the lesion criterion. Fourteen mice were successfully lesioned, giving a modeling success rate of 87.5%. Thirteen were used in the experimental modules, and one reserve animal was not included in data analysis. No deaths or exclusions occurred in the Sham group.

In the behavioral cohort assessed before treadmill intervention, net contralateral rotations differed significantly among groups (Sham: 7.40 ± 1.21; PD: 190.00 ± 8.79; PD+EX: 186.80 ± 5.89 turns/30 min; F(2,12) = 288.71, P = 7.12 × 10^-11^, η² = 0.980; **Figure 1F**). Holm-corrected comparisons showed greater rotation in the PD and PD+EX groups than in the Sham group (adjusted P = 6.52 × 10^-8^ and 5.21 × 10^-9^, respectively). Importantly, rotation did not differ between the PD and PD+EX groups before exercise (adjusted P = 0.7701), indicating comparable behavioral lesion severity before treadmill intervention.

TH immunofluorescence confirmed marked dopaminergic damage. Striatal TH intensity on the injected/lesioned side, expressed as a percentage of the contralateral side, was lower in PD mice than in Sham mice (Sham: 99.95 ± 0.94%; PD: 44.70 ± 1.20%; t(8) = 36.40, P = 3.56 × 10^-10^, Cohen’s d = 23.02; **Figures 1A, D**). SNpc TH intensity was also lower in the PD group (Sham: 100.51 ± 1.09%; PD: 46.92 ± 1.03%; t(8) = 35.85, P = 4.02 × 10^-10^, Cohen’s d = 22.67; **Figures 1B, E**).

Consistent with the immunofluorescence results, striatal TH protein normalized to β-actin was lower in PD mice than in Sham mice (Sham: 1.050 ± 0.071; PD: 0.355 ± 0.036; t(4) = 8.74, P = 0.0009, Cohen’s d = 7.14; **Figures 1C, G**). Together, these behavioral, histological, and biochemical findings confirmed successful establishment of the unilateral 6-OHDA lesion model.

### 3.2 Treadmill exercise improved motor performance in 6-OHDA-lesioned mice

**Figure 4:**
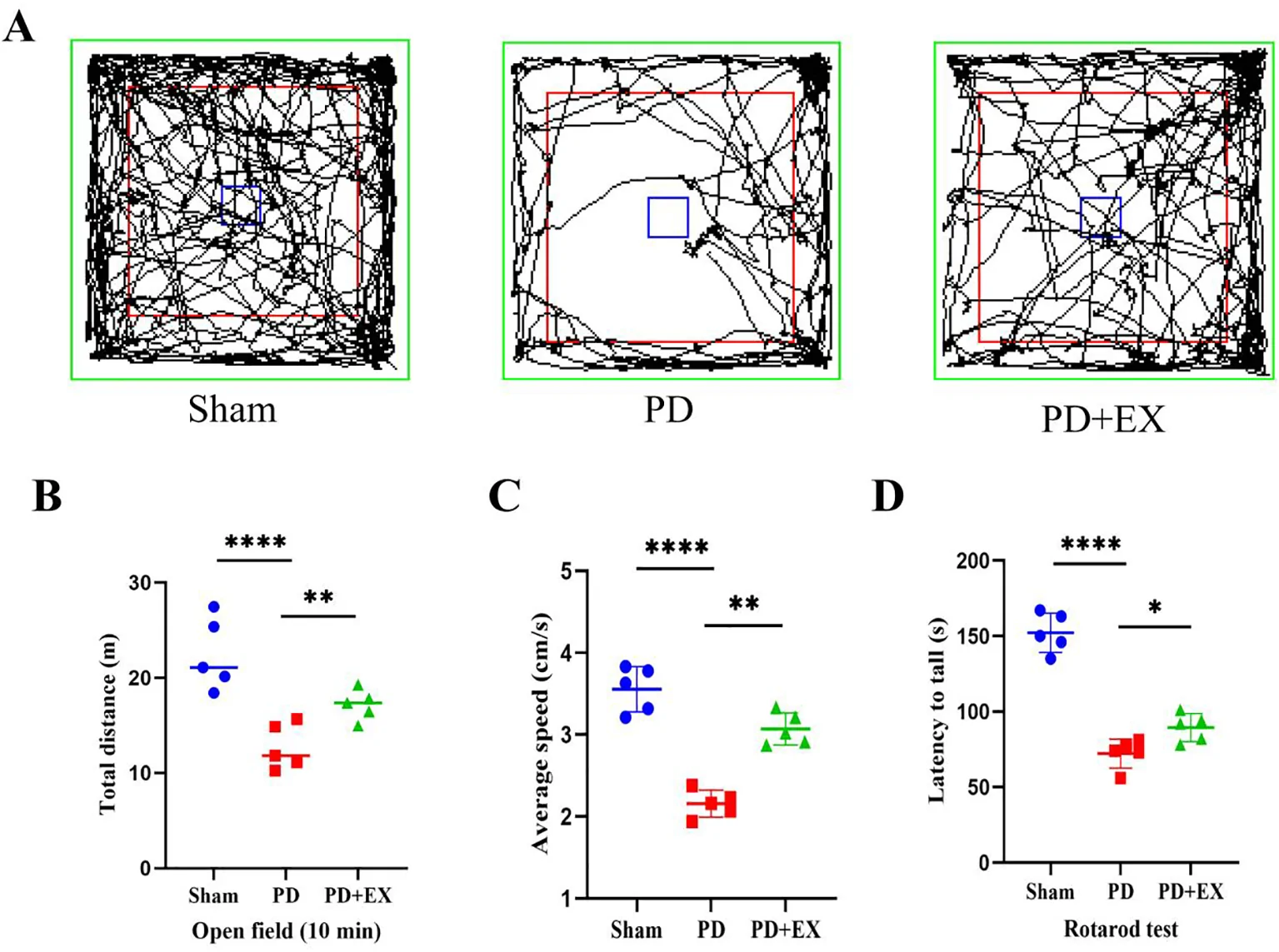
Treadmill exercise partially improved motor performance in 6-OHDA-lesioned mice. **(A)** Representative movement trajectories during the 10-min open-field test. The green boxes indicate the boundaries of the open-field arena, whereas the red and blue boxes indicate software-defined analysis regions. These boxes are shown only for trajectory visualization. **(B)** Total distance traveled during the open-field test. **(C)** Mean movement speed reported by the tracking software. **(D)** Latency to fall during the accelerating rotarod test. Each mouse underwent three formal rotarod trials, and the mean latency was used for analysis. Data are presented as mean ± SEM, n = 5 mice/group. Each animal was treated as an independent statistical unit. Comparisons were performed using one-way ANOVA followed by Holm-corrected pairwise comparisons. *P < 0.05, **P < 0.01, and ****P < 0.0001.

Representative open-field trajectories showed broadly distributed movement in Sham mice, restricted movement in PD mice, and an increased movement range in PD+EX mice (**Figure 4A**).

Total distance traveled differed significantly among groups (Sham: 22.50 ± 1.69 m; PD: 12.74 ± 1.07 m; PD+EX: 17.17 ± 0.71 m; F(2,12) = 15.90, P = 0.0004, η² = 0.726; **Figure 4B**). Total distance was lower in the PD group than in the Sham group (Holm-adjusted P = 0.0037) and higher in the PD+EX group than in the PD group (adjusted P = 0.0174). The PD+EX group remained lower than the Sham group (adjusted P = 0.0198), indicating partial rather than complete recovery.

Mean movement speed, as reported by the tracking software, also differed among groups (Sham: 3.55 ± 0.12 cm/s; PD: 2.16 ± 0.07 cm/s; PD+EX: 3.07 ± 0.09 cm/s; F(2,12) = 52.92, P = 1.12 × 10^-6^, η² = 0.898; **Figure 4C**). Mean movement speed was lower in the PD group than in the Sham group (adjusted P = 3.21 × 10^-5^) and higher in the PD+EX group than in the PD group (adjusted P = 9.36 × 10^-5^). The PD+EX group remained lower than the Sham group (adjusted P = 0.0126).

Rotarod latency differed significantly among groups (Sham: 152.20 ± 5.81 s; PD: 72.20 ± 4.28 s; PD+EX: 89.40 ± 4.17 s; F(2,12) = 76.61, P = 1.47 × 10^-7^, η² = 0.927; **Figure 4D**). Latency was shorter in the PD group than in the Sham group (adjusted P = 1.17 × 10^-5^) and longer in the PD+EX group than in the PD group (adjusted P = 0.0206). The PD+EX group nevertheless remained lower than the Sham group (adjusted P = 4.43 × 10^-5^).

These findings indicate that unilateral dopaminergic denervation substantially impaired spontaneous locomotion, motor coordination, and balance, whereas treadmill exercise partially restored motor performance. The consistent improvement in both open-field and rotarod outcomes supports recovery of functional motor capacity rather than merely an increase in exploratory behavior.

### 3.3 Regional PV protein abundance and basic membrane properties of SNr PV-lineage neurons

The SNr recording region and the identification of tdTomato-labeled PV-lineage neurons are shown in **Figures 2A, B**. A representative SNr section from a Pvalb-Cre;Ai14 mouse further showed qualitative colocalization between tdTomato-labeled PV-lineage cells and PV immunoreactivity within the SNr (**Figure 2D**). This image was included to provide representative cellular and anatomical context and was not used for quantitative colocalization analysis, cell counting, or between-group comparisons.

Regional PV Western blot analysis showed no significant difference in PV protein abundance among the Sham, PD, and PD+EX groups (Sham: 0.993 ± 0.024; PD: 0.977 ± 0.020; PD+EX: 0.977 ± 0.019; F(2,6) = 0.21, P = 0.8176, η² = 0.065; **Figures 2C, E**). Because these samples consisted of bulk lesioned-side nigral tissue containing the SNr, this result indicates only that no detectable group difference in overall regional PV protein abundance was observed. It does not establish that PV expression in individual PV-lineage neurons or the number of PV-positive neurons remained unchanged.

Whole-cell patch-clamp recordings included 9 cells from 5 Sham mice, 10 cells from 5 PD mice, and 9 cells from 5 PD+EX mice. Cell-level measurements were averaged within each animal before group-level analysis.

Resting membrane potential did not differ significantly among groups (Sham: −52.77 ± 1.14 mV; PD: −50.83 ± 0.78 mV; PD+EX: −51.53 ± 0.99 mV; F(2,12) = 0.99, P = 0.3996, η² = 0.142; **Figure 2F**). Membrane capacitance was also not significantly different among groups (Sham: 19.40 ± 1.77 pF; PD: 20.16 ± 1.46 pF; PD+EX: 20.60 ± 1.47 pF; F(2,12) = 0.15, P = 0.8634, η² = 0.024; **Figure 2G**).

Input resistance differed significantly among groups (Sham: 502.82 ± 8.43 MΩ; PD: 311.21 ± 23.35 MΩ; PD+EX: 408.31 ± 14.39 MΩ; F(2,12) = 33.44, P = 1.24 × 10^-5^, η² = 0.848; **Figure 2H**). Input resistance was lower in the PD group than in the Sham group (Holm-adjusted P = 0.0002) and higher in the PD+EX group than in the PD group (adjusted P = 0.0076). The PD+EX group remained lower than the Sham group (adjusted P = 0.0009), indicating partial normalization.

Action potential threshold also differed significantly among groups (Sham: −53.91 ± 0.89 mV; PD: −47.30 ± 1.06 mV; PD+EX: −51.28 ± 0.85 mV; F(2,12) = 12.60, P = 0.0011, η² = 0.677; **Figure 2I**). The threshold was more depolarized in the PD group than in the Sham group (adjusted P = 0.0042). It shifted toward the Sham level in the PD+EX group compared with the PD group (adjusted P = 0.0379), whereas the Sham and PD+EX groups did not differ significantly (adjusted P = 0.0651).

Thus, 6-OHDA lesioning was associated with reduced input resistance and a depolarized action potential threshold in SNr PV-lineage neurons. These abnormalities were partially normalized after treadmill exercise, whereas resting membrane potential and membrane capacitance were unchanged.

### 3.4 Treadmill exercise shifted evoked firing properties of SNr PV-lineage neurons toward Sham levels

**Figure 5:**
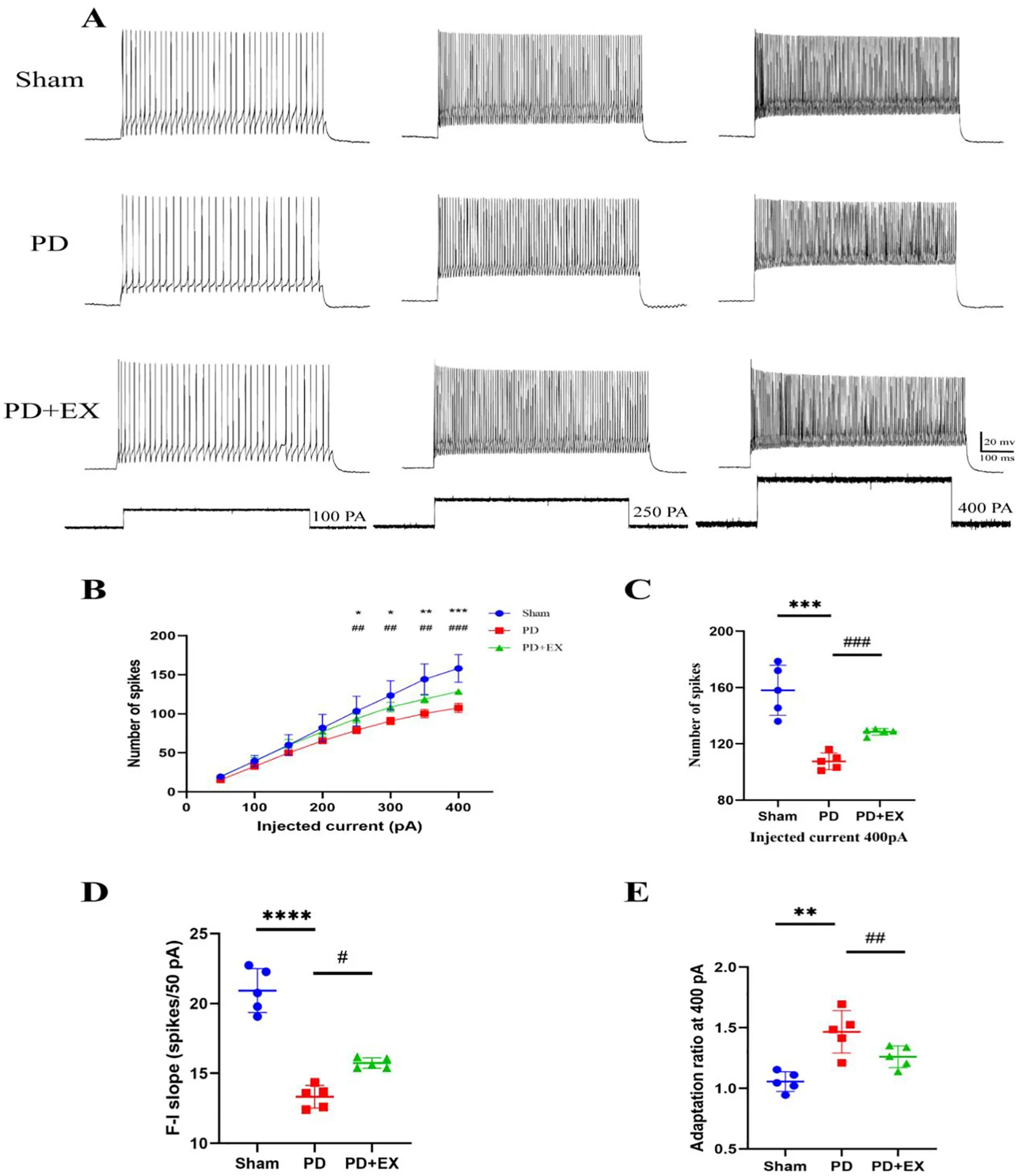
Treadmill exercise shifted the evoked firing properties of SNr PV-lineage neurons toward Sham levels. **(A)** Representative firing responses of SNr PV-lineage neurons from the Sham, PD, and PD+EX groups during 100-, 250-, and 400-pA depolarizing current injections. Voltage and time scale bars are shown in the lower-right corner, and corresponding current steps are shown below the traces. **(B)** Relationship between action potential number and injected current intensity. **(C)** Number of action potentials evoked by a 400-pA current injection. **(D)** Slope of the frequency–current **(F–I)** curve. **(E)** Firing adaptation ratio during a 400-pA current injection. The adaptation ratio was calculated as the last interspike interval divided by the first interspike interval; a higher value indicates greater firing adaptation. Patch-clamp recordings included 9 cells from 5 Sham mice, 10 cells from 5 PD mice, and 9 cells from 5 PD+EX mice. Values from multiple cells obtained from the same animal were averaged before group-level analysis, and the animal was treated as the independent statistical unit (n = 5 mice/group). Data are presented as mean ± SEM. The action potential number–current relationship in **(B)** was analyzed using two-way repeated-measures ANOVA, with group as the between-subject factor and current intensity as the within-subject factor, followed by Holm-corrected comparisons at individual current intensities. Data in **(C–E)** were analyzed using one-way ANOVA followed by Holm-corrected pairwise comparisons. In **(B–E)**, asterisks indicate Sham versus PD comparisons and number signs indicate PD versus PD+EX comparisons. *P or #P < 0.05; **P or ##P < 0.01; ***P or ###P < 0.001; ****P or ####P < 0.0001; ns, not significant.

Representative firing traces showed progressively increasing action potential output during stepwise depolarizing current injections in all groups (**Figure 5A**). Group differences became more apparent at the higher current intensities, with PD neurons showing the lowest firing output and PD+EX neurons displaying intermediate responses.

Two-way repeated-measures analysis showed significant effects of group (F(2,12) = 8.89, P = 0.0043, partial η² = 0.597), current intensity (F(7,84) = 1117.52, P = 4.72 × 10^-80^, partial η² = 0.989), and the group × current interaction (F(14,84) = 18.14, P = 9.44 × 10^-20^, partial η² = 0.751; **Figure 5B**).

For the primary comparisons, Holm-corrected tests showed that evoked firing was lower in the PD group than in the Sham group at 250, 300, 350, and 400 pA. Evoked firing was higher in the PD+EX group than in the PD group at the same current intensities. At lower current intensities, no consistent between-group differences were detected. Across the upper current range, the PD+EX values were intermediate between those of the Sham and PD groups, indicating a shift toward control-like firing rather than complete restoration.

At 400 pA, action potential number differed significantly among groups (Sham: 158.03 ± 7.95; PD: 107.57 ± 2.63; PD+EX: 128.53 ± 1.05; F(2,12) = 27.10, P = 3.55 × 10^-5^, η² = 0.819; **Figure 5C**). Action potential number was lower in the PD group than in the Sham group (adjusted P = 0.0006) and higher in the PD+EX group than in the PD group (adjusted P = 0.0002). The intermediate value in the PD+EX group was consistent with partial recovery of firing capacity.

The F–I curve slope also differed significantly among groups (Sham: 20.32 ± 1.07 spikes/50 pA; PD: 13.33 ± 0.36 spikes/50 pA; PD+EX: 15.74 ± 0.16 spikes/50 pA; F(2,12) = 29.24, P = 2.44 × 10^-5^, η² = 0.830; **Figure 5D**). The slope was lower in the PD group than in the Sham group and higher in the PD+EX group than in the PD group (both Holm-adjusted P < 0.05). Thus, treadmill exercise shifted the input–output gain toward the Sham level, although the group means remained separated.

The firing adaptation ratio differed significantly among groups (Sham: 1.243 ± 0.020; PD: 1.387 ± 0.016; PD+EX: 1.288 ± 0.017; F(2,12) = 17.21, P = 0.0003, η² = 0.742; **Figure 5E**). The adaptation ratio was higher in the PD group than in the Sham group (adjusted P = 0.0014) and lower in the PD+EX group than in the PD group (adjusted P = 0.0058), indicating improved maintenance of repetitive firing after exercise.

Collectively, unilateral 6-OHDA lesioning was associated with reduced evoked firing capacity, lower input–output gain, and greater firing adaptation in lesioned-side SNr PV-lineage neurons. Treadmill exercise shifted each of these measures toward the Sham level. Because the extent of recovery was not uniform across endpoints, the findings support partial functional normalization rather than complete restoration.

## 4 Discussion

The present study investigated whether the improvement in motor performance produced by treadmill exercise was accompanied by functional changes in a defined neuronal population of the SNr. Unilateral 6-OHDA lesioning caused marked rotational asymmetry, loss of TH signals in the striatum and SNpc, reduced spontaneous locomotion, and impaired rotarod performance. In parallel, PV-lineage neurons in the lesioned-side SNr showed lower input resistance, a depolarized AP threshold, reduced evoked firing and F-I gain, and greater firing adaptation. Six weeks of treadmill exercise improved the behavioral deficits and shifted several of these electrophysiological measures toward the Sham level. The cellular effects were incomplete, which is consistent with the partial rather than complete behavioral recovery.

Importantly, the PD and PD+EX groups showed comparable apomorphine-induced rotation before treadmill training, indicating similar behavioral lesion severity before the intervention. Previous studies have reported that treadmill exercise may attenuate nigrostriatal dopaminergic dysfunction in experimental parkinsonism. However, TH immunofluorescence and Western blot analyses in the present study included only the Sham and PD groups and were designed solely to verify successful lesion establishment. Therefore, we cannot determine whether the motor improvement observed in the PD+EX group involved preservation or restoration of the nigrostriatal dopaminergic system. Future studies should include TH-based histological and biochemical analyses in exercised animals to distinguish dopaminergic neuroprotection from circuit-level functional modulation.

Treadmill exercise increased open-field distance and speed and prolonged rotarod latency. The open-field findings indicate improved spontaneous locomotor output, whereas rotarod performance reflects coordination, balance, and sustained motor control. The agreement between the two tests suggests recovery of functional motor output rather than merely increased exploratory behavior and is consistent with previous reports of beneficial motor effects of aerobic exercise in PD models. Nevertheless, several behavioral values in PD+EX mice remained different from those of Sham mice, showing that training reduced but did not eliminate the consequences of the dopaminergic lesion. Electrical stimulation was not used to drive running, and sedentary animals received comparable handling. These procedures reduced nonspecific treatment differences, although forced treadmill exercise and repeated rotarod testing may still have induced stress or fatigue, which were not directly quantified in the present study.

The SNr occupies a strategic position in the basal ganglia because it integrates striatal, pallidal, and subthalamic input and regulates motor thalamic, collicular, and brainstem targets (Koketsu et al., 2021; McElvain et al., 2021; Ji et al., 2023; Aristieta et al., 2024; Thompson et al., 2025; Yoshida and Hikosaka, 2025). Dopamine depletion may therefore alter motor output not only by changing the balance of direct- and indirect-pathway input, but also by changing how individual SNr neurons transform input into firing (Zhou et al., 2009; Cáceres-Chávez et al., 2018; Cavarretta and Jaeger, 2023). In addition, somatodendritically released dopamine from SNpc neurons can directly excite SNr GABAergic neurons through D1/D5 receptor-dependent modulation of TRPC3 channels, suggesting that dopamine loss may directly influence SNr excitability through a local nigral pathway. The absence of significant changes in resting membrane potential and membrane capacitance suggests that the lesion did not produce a generalized disruption of all basic membrane properties. Instead, the principal differences involved input resistance and spike initiation. Lower input resistance would reduce the membrane voltage response generated by a given synaptic or injected current, whereas a more depolarized AP threshold would require greater depolarization before spike generation. These two changes provide a plausible cellular basis for the reduced firing output observed during depolarizing current injection.

The F-I analysis extends this interpretation beyond the response to a single current step. SNr PV-lineage neurons from PD mice generated fewer APs at higher current intensities, had a lower F-I slope, and showed greater adaptation during sustained depolarization. Thus, their ability to increase and maintain firing as input strength rose was reduced. Treadmill exercise shifted input resistance and AP threshold toward control values and improved both firing gain and firing maintenance. Because several measures remained different from Sham values, the data support partial functional normalization rather than complete restoration. This pattern also argues against describing the change simply as an overall increase or decrease in SNr excitability; the lesion affected specific components of input integration, spike initiation, and repetitive firing.

The present findings should be interpreted in the context of Delgado-Zabalza et al.^21, who previously demonstrated that 6-OHDA-induced parkinsonism reduces the intrinsic excitability of SNr PV-expressing neurons, is associated with downregulation of the sodium leak channel NALCN, and produces abnormal *in vivo* firing patterns. They further showed that chemogenetic manipulation of this neuronal population could improve motor performance. Therefore, the present study does not newly establish lesion-induced dysfunction of SNr PV-expressing neurons. Rather, it extends this previous work by showing that six weeks of treadmill exercise was accompanied by partial normalization of input resistance, action potential threshold, firing gain, and firing adaptation in genetically identified SNr PV-lineage neurons. Because NALCN expression and sodium leak currents were not measured in the present study, it remains unknown whether treadmill exercise influenced these electrophysiological properties through the same molecular mechanism (Delgado-Zabalza et al., 2023). These findings extend previous work showing that dopamine depletion weakens direct-pathway modulation of SNr neurons and that SNr GABAergic neurons are strongly shaped by intrinsic membrane conductances and synaptic integration (Barry et al., 2018; Gao et al., 2026). Previous electrophysiological studies in parkinsonian models have mainly examined overall SNr firing activity, synaptic inputs, or modulation of SNr neurons by the direct and indirect basal ganglia pathways (Cáceres-Chávez et al., 2018; Phillips et al., 2020; Sitzia et al., 2020; Aristieta et al., 2024; Shi et al., 2025). These studies have reported altered firing frequency, firing regularity, burst activity, and synaptic regulation after dopamine depletion. In contrast, the present study focused specifically on the intrinsic membrane properties and current-evoked firing capacity of genetically identified SNr PV-lineage neurons. Differences between the present findings and previous reports may reflect variation in neuronal subtype, lesion location and severity, recording configuration, experimental stage, and whether spontaneous network activity or intrinsic excitability was examined. Thus, our results complement rather than directly replicate previous studies by identifying cell-type-associated changes in input resistance, spike threshold, firing gain, and adaptation.

Exercise could influence these intrinsic properties through activity-dependent regulation of ion channels that determine membrane resistance, AP threshold, and spike-frequency adaptation. Exercise also modifies corticostriatal and thalamostriatal plasticity, neurotrophic signaling, oxidative balance, and neuroinflammatory activity (Chen et al., 2017; Chen et al., 2018; Bastioli et al., 2022). Changes in these upstream processes may alter the long-term synaptic environment of SNr neurons and promote homeostatic adjustment of their intrinsic membrane properties. The present experiments did not measure individual ion currents, synaptic transmission, neurotrophic factors, or inflammatory markers. These mechanisms are therefore biologically plausible explanations for the observed electrophysiological changes, but they were not directly tested.

No significant group difference was detected in PV protein abundance in bulk lesioned-side nigral samples. This negative result should be interpreted cautiously. The sampled region contained the SNr and adjacent nigral tissue, and Western blotting cannot isolate PV-lineage neurons or distinguish altered protein abundance per cell from changes in PV-positive cell number. Furthermore, Pvalb-Cre;Ai14 identifies cells with a history of Cre activity and does not guarantee PV protein expression at the experimental endpoint. Accordingly, the Western blot result is reported only as a regional PV protein measurement. It neither contradicts the electrophysiological findings nor demonstrates cell-specific regulation of PV expression.

The results should be interpreted within the scope of an exploratory study with modest animal numbers and no formal *a priori* power calculation. Averaging cell measurements within each mouse reduced the risk of pseudoreplication, but larger cohorts are needed to confirm the effect estimates. Only male mice were used to reduce potential variability associated with estrous-cycle-related hormonal fluctuations in this exploratory study. Nevertheless, this design prevents assessment of possible sex-dependent responses to 6-OHDA lesioning and treadmill exercise. The unilateral toxin lesion also limits extrapolation to bilateral pathology and the progressive, multisystem nature of human PD. Because the PD+EX group was not included in the TH assays, the contribution of dopaminergic preservation to behavioral improvement cannot be determined. In addition, the bulk regional Western blot provides no cell-specific information. Most importantly, the present findings demonstrate an association among treadmill exercise, improved motor performance, and altered electrophysiological properties of SNr PV-lineage neurons. Establishing a causal role for these neurons will require cell-type-specific manipulation and circuit-level experiments.

## 5 Conclusion

Six weeks of treadmill exercise improved spontaneous locomotion, coordination, and balance in unilateral 6-OHDA-lesioned mice. Motor improvement was accompanied by partial normalization of input resistance, AP threshold, evoked firing, F-I gain, and firing adaptation in lesioned-side SNr PV-lineage neurons. These results provide preliminary cellular electrophysiological evidence linking exercise intervention with functional modulation of a specific neuronal population in a basal ganglia output nucleus.

## Data Availability

The raw data supporting the conclusions of this article will be made available by the authors, without undue reservation.
